# Incidence and risk factors for disease and non-battle injury aboard the hospital ship USNS COMFORT during a Humanitarian Assistance and Disaster Response Mission, Continuing Promise 2011

**DOI:** 10.1186/s40794-016-0023-z

**Published:** 2016-04-27

**Authors:** Andy Chern, Andrea McCoy, Tracy Brannock, Gregory J. Martin, William T. Scouten, Chad K. Porter, Mark S. Riddle

**Affiliations:** 1 Uniformed Services University of the Health Sciences, 4301 Jones Bridge Road, Bethesda, MD 20814 USA; 2 Enteric Diseases Department, Naval Medical Research Center, 503 Robert Grant Avenue, Silver Springs, MD 20910 USA; 3 Air Force Global Strike Command, Barksdale Air Force Base, Bossier City, LA USA; 4 Department of State, Washington, DC USA; 5 Naval Medical Center Portsmouth, Portsmouth, VA USA

**Keywords:** Epidemiology, Humanitarian assistance, Disaster response, Occupational medicine, Military, Disease and non-battle injury, DNBI

## Abstract

**Background:**

Disease and non-battle injury (DNBI) are a leading cause of morbidity in deployments and can compromise operational mission performance. No study to date has examined DNBI incidence and impact aboard humanitarian aid/disaster response (HADR) mission ships.

**Methods:**

From April to September 2011, US military and civilian personnel participated in Continuing Promise 2011, a HADR training mission aboard USNS COMFORT (T-AH 20). Health surveillance was conducted for the purpose of assessing DNBI trends and improving force health protection during the deployment through passive surveillance, collection of DNBI data among those seeking care at the ship’s clinic, and actively through use of an anonymous weekly, self-report questionnaire. Categorical and total DNBI incidence rates were calculated per 100 person-weeks and incidence rate ratios (IRR) were calculated utilizing a negative binomial model to assess potential risk factors.

**Results:**

The leading syndrome-specific cause of weekly visits to the ship’s clinic was gastrointestinal (GI) followed by dermatologic and respiratory conditions (2.22, 1.97, and 1.46 cases per 100 person-weeks, respectively). The top three categorical DNBI were similarly represented by the questionnaire, with respiratory conditions having the highest reported rate followed by dermatologic and GI (11.79, 8.71, and 7.38 cases per 100 person-weeks, respectively). GI had the highest morbidity measures accounting for 61.9 % of lost work days and 27.3 % of reported moderate/severe impact to mission performance. Several factors were also associated with increased DNBI rates including personnel ages 26–36 (IRR = 1.23), officers (IRR = 1.23), days-off-ship (IRR = 1.09), and affiliation with nursing services (IRR = 1.48), naval mobile construction battalion (IRR = 3.17), and security (IRR = 1.71).

**Conclusions:**

DNBI can significantly impact mission performance on HADR missions, and establishing baseline rates and identifying risk factors can help improve force health protection in future HADR missions.

## Background

Disease and non-battle injuries (DNBI) can significantly impact operational missions and compromise readiness through loss of productivity and work days. Historically, DNBI have greatly outnumbered combat related injuries and led to considerable reduction in effectiveness. In World War II, the Korean War, and Vietnam conflict, DNBI accounted for over 60 % of non-effective rates, and even more recently during the initial phases of Operation Iraqi Freedom (OIF), 75 % of all hospitalizations were due to DNBI [1, 2]. Although DNBI can compromise readiness during combat deployments, DNBI also effect peacetime deployments. A few studies have even indicated higher DNBI rates during non-combat operations [3, 4].

Since 1997, the Department of Defense (DoD) has recognized the impact of DNBI on force health protection and mandated surveillance and reporting for DNBI among deployed units [5]. However, among these deployed units, not all DNBI are distributed similarly across different operational environments. A study of over 4000 deployed US Army soldiers during OIF showed that musculoskeletal injuries (e.g., sports/athletics and heavy gear/lifting) and psychiatric conditions encompassed nearly 75 % of the recorded DNBI [6]. Whereas, a study of the US Fifth Fleet found other medical/surgical conditions, dermatologic, and respiratory disease as the top three DNBI categories [7]. And, even aboard ships, several studies have found differential DNBI rates among different ship sizes [3, 4, 7].

The mandated surveillance and reporting by the DoD, however, only captures individuals actively seeking medical care in the clinic. Consequently, DNBI rates reported from clinic data underreport the true incidence of the diseases and injuries. Moreover, the percentage of those with DNBI seeking medical care varies across different DNBI categories. In studies among military personnel deployed to Iraq and Afghanistan, of those reporting infectious gastroenteritis, acute respiratory illness, and non-battle injury, only 22, 50 and 85 % (respectively) reported seeking medical care [8, 9].

Although previous studies have reported DNBI rates on US Navy ships during both combat and peacetime deployments, no study to date has examined DNBI rates aboard hospital ships engaged in humanitarian aid/disaster response (HADR) missions. Given the nature of HADR training and the operational mission of a hospital ship, the ship’s composition, consisting mostly of medical personnel, and their exposures are likely different than other Navy ships. Medical personnel, in particular, have a history of underreporting injuries and illnesses and have easier access to self-treatment [10–12].

Accounting for the unique population aboard HADR mission ships, the primary objectives of this study were to describe and compare total and categorical DNBI incidence rates from the ship’s clinic (passive) and survey questionnaire (active) surveillance data aboard a HADR mission ship, USNS COMFORT, during the Continuing Promise 2011 (CP 11) mission. Additionally, we aimed to determine potential risk factors among those that had a DNBI and sought medical care as well as describe morbidity measurements associated with categorical DNBI.

## Methods

The population in this study included all individuals serving aboard the USNS COMFORT during its 5-month CP 11 mission including uniformed service members (US and foreign) and non-governmental organization personnel. Weekly de-identified clinical aggregate data were compiled as mandated by the DoD for force health protection and public health surveillance for total and categorical DNBI including time aboard ship [5]. The case definitions for categorical clinic DNBI were defined in accordance with the DoD procedures for deployment health surveillance; specific case definitions are listed elsewhere [13–15]. Additionally, “combat/operational stress reactions” and “psychiatric, mental disorders” as defined by the DoD for deployment health surveillance were consolidated collectively under mental health conditions for analyses in this study. And, incidence rates for total and categorical DNBI, light duty, lost days, and admissions were calculated per 100 person-weeks. To further depict the burden of disease and injury, a graphical display was constructed plotting categorical DNBI incidence rates against categorical lost duty days per 100 categorical DNBI events.

An additional cross-sectional DNBI anonymous questionnaire utilizing a convenience sampling design was administered weekly to approximately 15 % (n ≈ 150) of the ship population to identify potentially uncaptured DNBI in the preceding week given that traditional DNBI data relies upon actively seeking healthcare. Questionnaires were administered to the available personnel reporting during morning muster or while awaiting transport to shore weekly. Each questionnaire identified basic demographics, departmental unit, days off the ship within the past week, seeking medical care within the past week (i.e. going to the clinic), ‘yes or no’ questions concerning categorical DNBI, and a question on DNBI impact on mission performance. Midway through the CP 11 mission, the questionnaire expanded the DNBI impact element beyond a dichotomous variable to produce additional granularity on impact to mission performance to better assist in force health protection.

Descriptive analyses were performed on demographic variables. Incidence rates for total and categorical DNBI from the survey were similarly calculated per 100 person-weeks. Negative binomial regression models were utilized to estimate unadjusted and adjusted relative incidence rate ratios (IRR) and to assess potential predictors of DNBI. Only statistically significant variables from univariate models were included in multivariate analyses (*p* < 0.05). Moreover, continuous variables (i.e., age and days-off-ship) and association with risk were tested for linearity and re-categorized if non-linear. Impact analyses utilized data after the questionnaire update previously described. Data from personnel indicating moderate and severe degree of impact were analyzed together to increase statistical power. Missing data on impact severity was excluded from analyses. Negative binomial modeling was also utilized to calculate IRR for those with DNBI seeking medical care. A negative binomial regression model was chosen due to the nature of the count data and to account for potential over-dispersion.

All analyses were performed with IBM SPSS Statistics 22 (IBM Corp. Released 2013. IBM SPSS Statistics for Windows, Version 22.0. Armonk, NY: IBM Corp), and applicable analyses operated under a two-tailed significance level set at *p*-values of less than 0.05.

## Results

During the 5-month CP 11 mission, approximately 900 personnel were serving aboard USNS COMFORT each week. Table 1 shows the demographic characteristics of personnel from clinic and the questionnaire. The weekly average number of men and women serving aboard the ship was 652 (72.2 %) and 251 (27.8 %), respectively. Among those administered the questionnaire, the median age was 29 (interquartile range [IQR] = 24–36 years) and there was slight oversampling of women (32.7 %) compared to the weekly average number of women aboard the ship (27.8 %). Personnel sampled from the questionnaire were predominantly affiliated with the Navy (79.3 %) followed by non-governmental organizations (6.8 %), which was similar to the average weekly distribution on the ship. Forty percent of the personnel completing the questionnaire reported their affiliation with a medically-related service (i.e. medical services, surgical services, and nursing services). Among those surveyed, there was no significant difference in demographic characteristics between those reporting a DNBI and those reporting no DNBI (data not shown). A total of 17,176 person-weeks (4779 female person-weeks) were represented from the clinic data, and a total of 3156 person-weeks (1031 female person-weeks) were represented from the questionnaire data.

**Table 1:** Demographics of personnel surveyed and serving aboard USNS COMFORT on CP11

	Ship population (weekly average)	Enhanced DNBI questionnaire
Gender		
Male	652 (72.2)	2038 (64.6)
Female	251 (27.8)	1031 (32.7)
Missing	N/A	87 (2.8)
Age		
Median [IQR]		29 [24–36]
≤ 25		976 (30.9)
26–35		1294 (41.0)
36–45		496 (15.7)
46–55		142 (4.5)
≥ 56		128 (4.1)
Missing		120 (3.8)
Service branch		
Army	11 (1.2)	71 (2.3)
Air Force	42 (4.7)	196 (6.2)
Navy	689 (76.6)	2503 (79.3)
NGO	54 (6.0)	213 (6.8)
Other	104 (11.6)	116 (3.7)
Missing	N/A	57 (1.8)
Rank		
Enlisted	569 (63.3)	1946 (61.7)
Officer	203 (22.5)	671 (21.3)
Civilian	127 (14.1)	269 (8.5)
Missing	N/A	270 (8.6)
Department		
Air detachment		128 (4.1)
Boat detachment		34 (1.1)
Department of Ancillary Services^a^		195 (6.2)
Destroyer Squadron		50 (1.6)
Department for Administration		291 (9.2)
Medical services		493 (15.6)
Surgical services		411 (13.0)
Nursing services		374 (11.9)
Maritime Expeditionary Security Squadron		93 (2.9)
Naval Mobile Construction Battalion		65 (2.1)
Manpower/Personnel		60 (1.9)
Security		315 (10.0)
Information operations		67 (2.1)
Communications		53 (1.7)
Public Affairs Office/Band		74 (2.3)
Translator		71 (2.2)
Operations		66 (2.1)
Other		316 (10.0)

^a^Department of Ancillary Services includes radiology, pharmacy, and laboratory

Data not obtained for age and department for ship population

Table 2 provides the categorical DNBI rates from both the ship’s clinic and the questionnaire surveillance data. The total weekly DNBI rate from clinic was 10.5 cases per 100 person-weeks. The DNBI categories of other medical/surgical, gastrointestinal infection, dermatologic and respiratory conditions were among the highest (3.18, 2.22, 1.97 and 1.46 cases per 100 person-weeks, respectively). In contrast, the questionnaire identified almost a 4 fold increase in the total DNBI rate compared to the clinic (42.2 versus 10.5 cases per 100 person-weeks). Respiratory, dermatologic, gastrointestinal infection and mental health conditions were among the highest identified in the questionnaire (11.79, 8.71, 7.38, and 5.29 cases per 100 person-weeks). Interestingly, the questionnaire identified a much lower rate for medical/surgical conditions compared to the clinic (0.76 versus 3.18 cases per 100 person-weeks, respectively), but at the same time, the questionnaire did uncover a greater than a 10 fold increase rate in mental health conditions compared to the clinic (5.29 versus 0.51 cases per 100 person-weeks, respectively).

**Table 2:** DNBI rates and impact measures from aggregate clinic visits and questionnaire during CP11

	Clinic visits		Clinic visits			Enhanced DNBI questionnaire		Enhanced DNBI questionnaire^bc^		
	Rate	95 % CI	Light duty days (%)	Lost work days (%)	Admissions (%)	Rate	95 % CI	No impact (%)	Minor impact (%)	Moderate/severe impact (%)
Respiratory	1.46	1.29, 1.65	13 (3.4)	39 (12.0)	1 (8.3)	11.79	10.63, 13.03	62 (26.7)	20 (13.5)	13 (16.9)
Dermatologic	1.97	1.77, 2.19	72 (18.8)	5 (1.5)	1 (8.3)	8.71	7.73, 9.79	69 (29.7)	32 (21.6)	4 (5.2)
Gastrointestinal	2.22	2.00, 2.45	13 (3.4)	201 (61.8)	3 (25.0)	7.38	6.48, 8.38	37 (15.9)	32 (21.6)	21 (27.3)
Gynecologic^a^	0.86	0.62, 1.15	0 (0.0)	2 (0.6)	0 (0.0)	0.87	0.43, 1.60	3 (1.3)	1 (0.7)	0 (0.0)
Heat injury	0.03	0.01, 0.07	0 (0.0)	1 (0.3)	0 (0.0)	1.87	1.44, 2.40	9 (3.9)	13 (8.8)	6 (7.8)
Sports/Recreation injury	0.13	0.08, 0.19	40 (10.4)	2 (0.6)	0 (0.0)	1.49	1.11, 1.96	12 (5.2)	5 (3.4)	2 (2.6)
MVA	0.00	0.00, 0.00	0 (0.0)	0 (0.0)	0 (0.0)	0.03	0.00, 0.16	0 (0.0)	0 (0.0)	0 (0.0)
Work/Training Injury	0.34	0.26, 0.44	58 (15.1)	12 (3.7)	0 (0.0)	0.98	0.68, 1.38	1 (0.4)	3 (2.0)	1 (1.3)
Other injury	0.17	0.12, 0.24	47 (12.3)	3 (0.9)	0 (0.0)	0.86	0.58, 1.23	6 (2.6)	5 (3.4)	2 (2.6)
Ophthalmologic	0.13	0.09, 0.20	0 (0.0)	3 (0.9)	0 (0.0)	1.08	0.76, 1.49	3 (1.3)	3(2.0)	4 (5.2)
STI	0.03	0.01, 0.07	0 (0.0)	0 (0.0)	0 (0.0)	0.03	0.00, 0.16	0 (0.0)	0 (0.0)	0 (0.0)
Mental health	0.51	0.41, 0.62	0 (0.0)	1 (0.3)	2 (16.7)	5.29	4.53, 6.14	22 (9.5)	28 (18.9)	11 (14.3)
Fever	0.02	0.00, 0.05	0 (0.0)	0 (0.0)	0 (0.0)	1.52	1.13, 2.00	5 (2.2)	4 (2.7)	9 (11.7)
Other Med/Surgical	3.18	2.92, 3.45	140 (36.6)	49 (15.1)	4 (33.3)	0.76	0.50, 1.11	3 (1.3)	2 (1.4)	4 (5.2)
Dental						0.10	0.02, 0.26	0 (0.0)	0 (0.0)	0 (0.0)
Neurologic	0.10	0.06, 0.16	0 (0.0)	3 (0.9)	1 (8.3)					
Total	10.53	10.05, 11.03	383 (100.0)	325 (100.0)	12 (100.0)	42.17	39.95, 44.48	232 (42.1)	148 (26.9)	77 (14.0)

Data not obtained for clinic dental or questionnaire neurologic

Rates are in cases per 100 person-weeks

^a^Gynecologic rates calculated using female person-time

^b^Impact analysis accounting for periods after implementing updated questionnaire at week 13

^c^17 % with a DNBI did not indicate any degree of impact

*CI* confidence interval, *MVA* motor vehicle accident, *STI* sexually transmitted infection

**Fig. 1:**
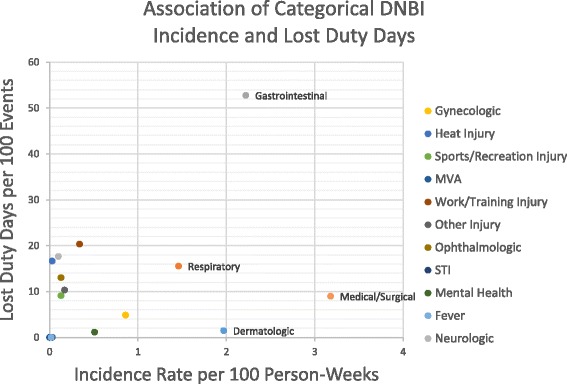
DNBI Incidence rates and lost work days from clinic data aboard USNS COMFORT during CP11. Events denote corresponding categorical DNBI

During the mission, the ship’s clinic captured light duty days, lost days, and admissions at a rate of 2.23, 1.89, and 0.07 per 100 person-weeks, respectively (data not shown in table). The majority of light duty days were attributed to other medical/surgical conditions (140/383, 36.6 %), which was also the leading cause for clinic visits (546/1809, 30.2 %). Gastrointestinal infections were the primary DNBI leading to lost work days (201/325, 61.8 %) and were the second leading cause for clinic visits (381/1809, 21.1 %). The relationship between lost work days and DNBI incidence is depicted in Fig. 1, which shows a significant burden from gastrointestinal infections. Personnel answering the questionnaire also corroborated this result indicating that gastrointestinal infections were also the primary DNBI that had the most adverse impact on their mission performance (Table 2). Gastrointestinal conditions accounted for 21.6 (32/148) and 27.3 (21/77) percent of those reporting minor and moderate/severe impacts, respectively. Respiratory conditions followed gastrointestinal infections as the second leading DNBI causing moderate/severe degree of impact to mission performance, accounting for 12 % (39/325) of all lost work days.

Table 3 displays the number of personnel reporting and seeking care for DNBI as well as the differential DNBI counts based on the questionnaire. Overall, the questionnaire identified 29.2 % (921/3156) of respondents that self-reported at least one DNBI. However, only 16.3 % (150/921) reporting a DNBI sought medical care from the ship’s clinic. Respiratory and gastrointestinal conditions were the most common DNBI associated with seeking medical care (44 %, 113/257). However, personnel reporting a medical/surgical condition or fever had the highest proportion within a DNBI category to seek medical care (54.2 and 45.8 %, respectively).

**Table 3:** Personnel reporting DNBI and seeking medical care from questionnaire with differential DNBI counts from CP11

	Reported DNBI	Reported DNBI and sought medical care	Total
Number of personnel	921	150	3156
DNBI	DNBI count	Number seeking care	Percentage (%)
Respiratory	372	59	15.9
Dermatologic	275	41	14.9
Gastrointestinal	233	54	23.2
Gynecologic	9	3	33.3
Heat Injury	59	13	22.0
Sports/Recreation Injury	47	12	25.5
MVA	1	0	0.0
Work/Training Injury	31	9	29.0
Other Injury	27	7	25.9
Ophthalmologic	34	9	26.5
STI	1	0	0.0
Mental Health	167	14	8.4
Fever	48	22	45.8
Medical/Surgical	24	13	54.2
Dental	3	1	33.3
Total	1331	257	19.3

Personnel may have reported ≥ 1 DNBI

Table 4 displays the results of the univariate and multivariate analyses. Of note, age was initially divided into 6 categories, but due to a lack of linearity was re-categorized into 3 age groups. Days-off-ship was kept as a continuous variable based on graphical and quadratic analyses.

**Table 4:** Univariate and multivariate analyses of DNBI rates among surveyed personnel aboard USNS COMFORT during CP11

	Total DNBI rates			
	Univariate		Multivariate^a^	
	IRR	*p*-value	IRR	*p*-value
Age				
≤ 25 (referent)	1.00		1.00	
26–35	1.17	0.049	1.23	0.009
36+	0.87	0.133	0.90	0.316
Gender				
Male (referent)	1.00			
Female	1.00	0.977		
Rank				
Enlisted (referent)	1.00		1.00	
Officers	1.07	0.440	1.23	0.025
Civilian	0.76	0.024	0.93	0.934
Branch of service				
Army (referent)	1.00			
Air Force	1.60	0.144		
Navy	1.65	0.087		
NGO	1.30	0.412		
Other	1.74	0.089		
Days off Ship (continuous)	1.10	<0.001	1.09	<0.001
Location				
Jamaica	0.75	0.036	1.07	0.672
Peru	0.70	0.025	1.03	0.889
Ecuador	1.60	<0.001	1.81	<0.001
Colombia	1.39	0.001	1.39	0.002
Nicaragua	1.13	0.366	1.61	0.002
Guatemala	1.24	0.072	1.64	<0.001
El Salvador	1.60	<0.001	2.06	<0.001
Costa Rica	0.81	0.072	1.10	0.472
Haiti	0.52	<0.001	0.84	0.270
Unit department				
Air detachment	1.29	0.127	1.45	0.062
Boat detachment	0.70	0.316	0.93	0.837
DAS	0.82	0.166	1.05	0.795
Destroyer Squadron	0.71	0.278	0.75	0.374
DFA	0.97	0.805	1.11	0.539
Medical services	1.00	0.996	1.17	0.268
Surgical services	0.74	0.003	0.96	0.790
Nursing services	1.29	0.004	1.48	0.007
Maritime Expeditionary Security Squadron	0.73	0.286	1.13	0.698
Naval Mobile Construction Battalion	2.25	<0.001	3.17	<0.001
Manpower/Personnel	0.83	0.453	1.20	0.521
Security	1.27	0.025	1.71	<0.001
Information operations	0.63	0.062	0.71	0.210
Communications	1.17	0.546	1.39	0.247
Public Affairs Office/Band	1.29	0.174	1.35	0.164
Translator	0.90	0.665	1.01	0.969
Operations	0.93	0.799	1.16	0.589
Other	0.74	0.016	0.80	0.061

^a^Multivariate analyses adjusted for age, rank and days off ship

In univariate analyses, we found an increased rate of DNBI in personnel ages 26–35 (IRR = 1.17, *p* = 0.049) compared to the youngest age category, and civilians had a lower rate of DNBI (IRR = 0.76, *p* = 0.024) compared to enlisted. We also observed a 10 % increase in DNBI rate for each additional day reported off the ship (IRR = 1.10, *p* < 0.001). Additionally, mission locations to Jamaica, Peru and Haiti had lower DNBI rates (IRR = 0.75, 0.70 and 0.52, respectively), whereas, missions to Ecuador, Colombia and El Salvador had higher rates (IRR = 1.60, 1.39 and 1.60, respectively) relative to all other mission locations. Personnel in the surgical services had a lower rate of DNBI, while those in nursing, naval mobile construction battalion, and security all had higher DNBI rates compared to all other departmental units. The only variable associated with seeking medical care was the degree of impact on mission performance in an apparent dose–response relationship. Respondents reporting minor and moderate/severe impacts were 3.67 and 5.90 times more likely to seek medical care compared to those reporting no impact to mission performance from their DNBI (data not shown).

After controlling for important covariates, personnel between the ages of 26–35 and days spent off the ship retained significance. Officers had a 1.23 times greater risk of DNBI compared to the enlisted ranks (*p* = 0.009). Mission locations in Ecuador, Colombia, Nicaragua, Guatemala and El Salvador had increased IRR ranging from 1.39 to 2.06. The low IRR for personnel in the surgical services no longer was significant. Nursing, naval mobile construction battalion and security, however, continued to have greater IRR compared to all other services (IRR = 1.48, 3.17 and 1.71, respectively).

## Discussion

This study provides the first baseline assessment of the incidence and impact of DNBI specifically aboard a US Navy hospital ship participating in a HADR training mission. Consistent with prior DNBI studies, our data shows that DNBI are underreported [8, 9, 16]. The overall clinic DNBI rate was 10.05 cases per 100 person-weeks, but our questionnaire identified a greater than 4 fold increase in the DNBI rate at 42.17 cases per 100 person-weeks. The overall clinic-based DNBI rate was also ≥ 2 times higher than the rates observed in two non-humanitarian shipboard studies (DNBI rates between 4.1 and 4.4 cases per 100 person-weeks) [7, 17]. In contrast, the rate of admissions during the CP 11 mission (0.07 admission per 100 person-weeks) was similar to the rate of a non-humanitarian U.S. Fleet during peacetime deployment (0.06 admission per 100 person-weeks) [17].

Our study also showed a relatively low proportion of personnel seeking medical care compared to previous DNBI studies. In two separate studies of service members deployed to Iraq and Afghanistan, the percentage of those reporting non-battle injuries (i.e., sports/athletics, heavy gear/lifting, fall, machinery/tools, vehicle accident, other) that sought medical care ranged from 77 to nearly 85 % compared to no greater than 29 % for any injury category in our study [9, 16]. Even among the specific gastrointestinal and respiratory diseases, the rates we observed were on the lower end of those seen in other studies. The rates for seeking medical care from our study were 23.2 and 15.9 %, respectively, where other studies of personnel deployed to southwest Asia and the Middle East ranged from 22.4 to 48.3 % for diarrheal diseases and 17 to 50 % for respiratory diseases [8, 16]. These lower rates for gastrointestinal infection were even more surprising as there were frequent health threat briefs and active messaging for people to seek care for diarrhea and/or vomiting (*Riddle, personal communication*). While it is unclear why we observed lower rates of seeking medical care among those with a DNBI, one explanation could be the different composition of personnel aboard the ship. Given the relatively large proportion of medical providers on the ship, informal medical care may have been common. In a survey study by Gendel and colleagues, 97 % of physicians surveyed reported providing informal care (e.g., prescribing without an official clinic visit) for minor conditions, and as many as 59 % of physicians reported providing informal care even to the degree of treating chronic or serious medical conditions [13]. However, we did not observe a lower rate of seeking medical care among any of the medical services potentially indicative of receiving informal medical care from medical colleagues. Nonetheless, increased study of the potential for informal care and its impact on DNBI surveillance is needed to better understand this potential phenomenon.

The morbidity measurements in our study indicate underreporting even when the DNBI caused some degree of impact to mission performance. Among those reporting a DNBI on the questionnaire, 36 % reported some degree of impact while only 10 % of these individuals reported seeking medical care. In fact, among the leading categorical DNBI reported as having the most significant impact (i.e., gastrointestinal, respiratory, dermatologic, and mental health), more individuals did not seek care compared to those that did across all impact categories except for respiratory, where those reporting moderate/severe degree of impact were more equally likely to seek care or not seek care. As noted previously, 17 % of individuals reporting a DNBI did not indicate any impact (i.e., no impact, minor impact, moderate/severe impact) with the updated questionnaire, which may have skewed the categorical DNBI identified as having the most severe effects on mission performance. The majority of missing data for DNBI impact came from respiratory, dermatologic and gastrointestinal conditions, which accounts for nearly 70 % of the missing data. However, given that most of the missing data were attributed to those that did not seek medical care, our data still shows that there is significant morbidity from DNBI that is underreported, and perhaps, encouraging self-referral for some of these more common DNBI early may help reduce the impact to overall performance and mission.

A number of factors were associated with increased rates of DNBI. Compared to the youngest age group (≤25), respondents 26–35 years old had a higher DNBI rate, an effect not observed in the oldest age group (˃ 35). Prior DNBI studies have been mixed showing either no difference or a higher DNBI rate with increasing age [8, 9, 16]. Additionally, prior studies also have shown mixed results for the association between rank and DNBI rates, leading some authors to hypothesize that the increased DNBI rates seen in enlisted personnel may be partly attributed to participation in more hazardous activities [9]. However, we did not observe these findings as our multivariate model showed officers having a higher DNBI rate than the enlisted (IRR = 1.23, *p* = 0.025).

We also found differential risk based on mission location. Ecuador (IRR = 1.81) and El Salvador (IRR = 2.06) had the highest DNBI rates relative to all other locations. This increased risk of DNBI may be partly attributed to the liberty periods while in Ecuador and to differences in environmental exposures in El Salvador. Particularly for gastrointestinal infections, one of the leading symptom-specific DNBI reported in the questionnaire, the mission locations to Central and South American countries are considered high-risk areas for travelers’ diarrhea [18]. Further analyses of food consumption within a host country in relation to symptoms may help discern this potential association. The differential DNBI rates seen among the different countries are unlikely due to the economic status of the host countries given that most mission locations were upper middle and lower middle income countries, and the only low income country, Haiti, did not show any difference in DNBI rates (*p* = 0.270) [15]. Differential rates of DNBI more likely correlated to the exposure of personnel to local foods, which was variable during this deployment. The positive association found between increased rates of DNBI and number of days off the ship (IRR = 1.09, *p* < 0.001) supports this finding. It is also important to take into consideration the incubation time for gastrointestinal and respiratory diseases, which were among the top DNBI. Although increased rates were found at specific mission locations, the actual exposure may have been in the prior host country.

Occupation was also associated with differential risk. Service members in nursing services, the construction battalion, and security had the highest risk. A variety of physical and biologic hazards are recognized in nursing related services including ergonomic and lifting injury risks as well as infectious disease exposure, and their close contact with patients often place them at higher risk of occupational exposures compared to other healthcare workers [19]. This may, in part, explain some of the increase in the DNBI rate. The other high risk occupations identified frequently had off-ship exposures and access to non-ship food.

Although our study provides a solid baseline for future hospital ship-based HADR training missions, we also recognize limitations. As with many survey questionnaires that refer to past events, recall bias is a possibility. Furthermore, the questionnaire could have been susceptible to misclassification of categorical DNBI (e.g., sore throat from a respiratory versus a gastrointestinal etiology). We attempted to limit these factors by distributing a weekly survey asking only about events in the preceding week and asking about specific symptoms associated with different categorical DNBI. The survey questionnaire also utilized a convenience sampling method, which is prone to selection bias (healthy worker effect), though the distribution was similar to the overall ship’s population based on demographics.

## Conclusions

This study provides the first assessment of DNBI rates and associated impact aboard a US Navy hospital ship-based HADR training mission. We found increased total DNBI rates as well as a low proportion of personnel seeking medical care compared to prior studies. In addition, we found increased DNBI rates associated with several variables (i.e., age, rank, days off ship, unit department). Future studies should confirm and refine these associations. Using DNBI data, investigators should devise strategies for high risk populations and environments that mitigate the detrimental effects of DNBI on mission performance.

### Ethics approval and consent to participate

The data were collected for public health surveillance and monitoring and were not under a research protocol. This analysis and reporting of these data were determined to be exempt from human subjects research by the Uniformed Services University of the Health Sciences Institutional Review Board.

### Consent for publication

This study does not contain any personally identifiable information.

### Availability of data materials

The datasets supporting the conclusions of this article are available through the Naval Medical Research Center in Silver Springs, Maryland, by special request.

## Abbreviations

CP 11: continuing promise 2011
DNBI: disease and non-battle injury
DoD: Department of Defense
GI: gastrointestinal
HADR: humanitarian aid/disaster response
IQR: interquartile range
IRR: incidence rate ratio
USNS: United States Naval Ship
